# Examining the relationships of happiness and emotional symptoms, regular exercise and demographic characteristics among adolescents seeking psychological services: cross-sectional study with mediation analysis

**DOI:** 10.1192/bjo.2025.22

**Published:** 2025-03-31

**Authors:** Na Yin, Jing Zhang, Honghong Ren, Bin Wang, Guoqing Zhao, Yumei Du

**Affiliations:** Department of Traditional Chinese Medicine, Shandong Provincial Hospital Affiliated to Shandong First Medical University, Jinan, Shandong, China; Department of Traditional Chinese Medicine, Shandong Provincial Hospital, Shandong University, Jinan, Shandong, China; Psychiatry Department, Shandong Daizhuang Hospital, Jining, Shandong, China; Department of Psychology, Shandong Provincial Hospital Affiliated to Shandong First Medical University, Jinan, Shandong, China; Department of Psychology, Shandong Provincial Hospital, Shandong University, Jinan, Shandong, China; Department of Healthycare Respiratory, Shandong Provincial Hospital Affiliated to Shandong First Medical University, Jinan, Shandong, China; Department of Healthycare Respiratory, Shandong Provincial Hospital, Shandong University, Jinan, Shandong, China

**Keywords:** Adolescents, happiness, physical exercise and dietary habits, anxiety and depression symptoms, parental marital quality

## Abstract

**Background:**

Adolescence marks a critical transition period, with significant mental health challenges including anxiety and depression symptoms that affect long-term happiness. There has been a lack of research exploring the factors mediating adolescent happiness.

**Aims:**

To investigate the mediating effects of anxiety and depression on adolescent happiness, as well as the contributions of sociodemographic factors.

**Methods:**

We recruited 392 adolescents. Anxiety symptoms, depression symptoms and happiness were assessed by the seven-item Generalized Anxiety Disorder scale, nine-item Patient Health Questionnaire and single-item happiness scale, respectively. Self-administered questionnaires were used to collect sociodemographic information.

**Results:**

Spearman correlation analysis showed significant negative correlations of happiness with anxiety (*r* = −0.37, *P* < 0.0001) and depression (*r* = −0.47, *P* < 0.0001). Positive predictors of happiness included quality of parents’ marriage (*β* = 0.12, *P* = 0.006), regular physical exercise (*β* = 0.13, *P* = 0.006) and regular diet (*β* = 0.10, *P* = 0.03). Mediation analysis indicated that depressive symptoms (estimate = 0.50, 95% CI: 0.25 to 0.80) and anxiety symptoms (estimate = 0.32, 95% CI: 0.12 to 0.57) partially mediated the relationship between regular exercise and happiness, whereas depressive symptoms completely mediated the relationship between anxiety symptoms and happiness (estimate = −0.14, 95% CI: −0.20 to −0.08).

**Conclusion:**

The findings of this study highlight the intricate interplay of mental health issues, lifestyle factors and adolescent happiness and emphasise the need for comprehensive interventions focusing on enhancing physical activity and addressing psychological health to foster happiness among adolescents.

Adolescence represents a crucial period of transition from childhood to adulthood and is marked by the establishment of lifestyle behaviours that significantly affect both immediate and long-term health outcomes.^1^ During this time, adolescents face numerous challenges, notably a high prevalence of mental disorders. Worldwide, as many as 20% of adolescents struggle with mental health issues,^2^ predominantly anxiety and depression.^3^ These conditions often co-occur and affect 25 to 50% of this demographic,^4^ leading to significant personal and familial distress, as well as serious repercussions including academic underperformance, cognitive impairments, strained interpersonal relationships and increased risk of suicide.^5^ The extension of these conditions into adulthood^6^ adds to the urgent need for early interventions.

## Happiness in adolescence: public health imperative

Subjective happiness, which is a nuanced concept typically segmented into overall life evaluations and emotional states,^7^ has emerged as a significant focus in public policy over recent decades.^8^ Insufficient levels of subjective happiness during adolescence can result in behavioural and social difficulties, lowered self-esteem, loneliness, poor academic outcomes and family dysfunction,^9^ further compounding mental health problems.^10^ In addition, as adolescents grow older, they undergo a noticeable decline in positive mental health, with older teenagers experiencing reduced happiness and more severe depressive symptoms compared with their younger counterparts.^11^ This trend indicates a critical need to prioritise factors that enhance happiness during these formative years to sustain happiness and prevent long-term adverse outcomes. In summary, happiness, as a positive emotional state, serves as a crucial indicator of an adolescent’s psychological and emotional health, quality of life and even potential for future success.^12^ Evidence suggests that sufficient happiness during youth can predict healthy behaviours and subsequent health outcomes. Given the strong correlations of happiness with both the current and future physical and mental health of the population, assessing and enhancing happiness should be prioritised as part of the public health agenda.

## Physical exercise: mediating mental health and happiness

Regular physical exercise is recognised as a vital protective factor for mental health, particularly in adolescents engaged in sporting activities.^13^ Research has revealed complex relationships among physical exercise, emotional disorders (such as anxiety and depression) and happiness. Specifically, exercise can indirectly enhance happiness by reducing anxiety and depression.^14^ This mediating effect model illustrates how exercise improves individuals’ happiness through the enhancement of emotional states. For example, regular physical activity can enhance the transmission of monoamines in the brain, such as norepinephrine, dopamine and serotonin, while also increasing levels of endorphins.^15^ Notably, studies also indicate that there may be bidirectional influences among happiness, emotional disorders and physical exercise.^16^ Given the complexity and uncertainty of these relationships – especially regarding whether anxiety and depression have the same role in the dynamic changes that occur – there is a need for more detailed analyses to identify the different parts that anxiety and depression may play in various situations and clarify their specific functions in the mediation process. Furthermore, research has shown correlation between happiness and social demographic factors and individual habits.^17^ This indicates a significant relationship between happiness and social demographic factors that deserves attention as part of public health intervention measures.

To our knowledge, comprehensive mediation analyses exploring the intermediary factors linking regular physical exercise, emotional disorders and happiness remain scarce. Previous studies of these relationships have predominantly focused on student populations, with limited attention to clinical groups. Adolescents seeking psychological services face more severe mental health challenges, demonstrate greater awareness of mental health issues and are more likely to adopt proactive coping strategies. Recognising these distinctions, in this study, we investigated the complex relationships among adolescent happiness, anxiety, depression and regular exercise through mediation analysis. By focusing on this unique population, we aimed to elucidate the intricate interdependencies among these factors and provide novel insights into pathways that enhance happiness.

## Method

A cross-sectional study design was used to analyse data collected from the Clinical Psychology Outpatient Clinic at Shandong Provincial Hospital in Jinan, China. Adolescents seeking psychological services were recruited through advertisements. After guardian consent had been obtained, these participants voluntarily signed an informed consent form and completed a series of online surveys before accessing psychological services. All self-administered questionnaires were completed under the guidance of specialised psychiatrists, ensuring that participants received appropriate support and guidance.

### Participants

Between June 2021 and May 2022, we initially recruited 592 participants. Participants were excluded if they were less than 12 years old (*n* = 4), more than 18 years old (*n* = 43), had a 32-item Hypomania Checklist score of 13 or higher (*n* = 113) or Mood Disorder Questionnaire score of 6 or higher (*n* = 25), belonged to a minority group (*n* = 6), were an elementary school student (*n* = 6) or had missing variables (*n* = 3). Consequently, 392 participants were included in the final analysis.^18^

Eligible adolescents met the following criteria: (a) Han ethnicity and right-handed, aged between 12 and 17 years; (b) actively seeking psychological services at the clinic; (c) free from physical illnesses in the past 3 months; (d) no history of alcohol or drug misuse or dependence, excluding tobacco; (e) no history of manic or hypomanic episodes; and (f) had not participated in online teaching during the recent coronavirus disease 2019 (COVID-19) pandemic wave.

### Sociodemographic information

Sociodemographic information, including age, sex, education level (junior or senior high school), sibling status (only child or not), residence (rural or urban), parents’ marital quality (poor, fair or good), general health status (poor, fair or good), regular exercise habits (yes or no), dietary practices (regular or not), history of mental illness (yes or no), history of somatic diseases (yes or no) and dietary preferences (vegetarian, balanced or predominantly meat-based), was collected through a self-designed questionnaire completed by adolescents.

### Clinical assessment

The 32-item Hypomania Checklist and the Mood Disorder Questionnaire were used to exclude the possibility of bipolar disorder in adolescents,^19^ and the nine-item Patient Health Questionnaire (PHQ-9) and seven-item Generalized Anxiety Disorder (GAD-7) scale were used to assess the severity of depressive and anxiety symptoms. Research in Chinese populations has indicated that the Chinese versions of the PHQ-9 and GAD-7 have strong reliability and validity.^20^

#### Anxiety symptoms

The GAD-7 scale is designed to assess anxiety levels, particularly in adolescents, although it is also applicable to adults.^21^ This scale includes seven items, each with a four-point Likert scale for responses: ‘Not at all’ (0), ‘Several days’ (1), ‘More than half the days’ (2) and ‘Nearly every day’ (3). The total score for the GAD-7 can range from 0 to 21, with higher scores reflecting greater anxiety. Specific cut-off points at scores of 5, 10 and 15 are used to indicate mild, moderate and severe anxiety levels, respectively.

#### Depressive symptoms

The PHQ-9 is a widely used diagnostic tool for assessing the severity of depressive symptoms in adults.^22^ It consists of nine items, each scored from 0 to 3, which correspond to the frequency of symptoms experienced over the past 2 weeks. The scoring options are: ‘Not at all’ (0), ‘Several days’ (1), ‘More than half the days’ (2) and ‘Nearly every day’ (3). The total possible score ranges from 0 to 27, with higher scores indicating more severe depressive symptoms. The thresholds for PHQ-9 are set at scores of 5, 10, 15 and 20, representing mild, moderate, moderately severe and severe depression, respectively.

#### Happiness

Recent research on adolescent happiness has used various assessment tools, including the Subjective Happiness Scale, the Oxford Happiness Questionnaire and the Oxford Happiness Inventory, in addition to numerous adaptations of single-item questions aimed at gauging current happiness levels.^23^ However, although these instruments are well-established, their ability to directly measure happiness has been questioned. For instance, the five-item version of the World Health Organization Well-Being Index does not explicitly assess happiness itself, focusing instead on broader well-being constructs.^24^ In this context, the 0-to-10 linear scale has emerged as a strong alternative.^25^ This straightforward measure allows respondents to easily indicate their happiness without the need for complex questionnaires or multiple items.^26^

A key advantage of the 0-to-10 scale is its practical utility in situations requiring quick assessment, such as during the COVID-19 pandemic, when reduced contact time was essential. Moreover, its simplicity and ease of understanding make it highly adaptable to different contexts, including community surveys and cross-cultural studies.^27^ Research has shown that a single item, such as the question ‘Do you feel happy in general?’ on an 11-point scale, provides a reliable and valid measure of happiness; strong temporal stability was demonstrated with a coefficient of 0.86, indicating a consistent ability to measure happiness over time.^28^

### Statistical analysis

All statistical analyses were performed using SPSS software (version 26.0, IBM Corp., Armonk, New York, USA), with a two-tailed significance threshold set to *P* = 0.05. Data normality was assessed before analysis using the Kolmogorov–Smirnov test. Categorical variables were represented using counts and percentages, whereas variables with skewed distributions were described using interquartile ranges. Spearman’s correlation analysis was used to examine relationships between variables, and *P*-values were adjusted using Bonferroni correction, establishing a significance threshold of *P* < 0.05/13 = 0.004. Variables demonstrating significant relationships in the correlation analysis were subsequently included in a stepwise multivariate linear regression analysis to identify factors influencing adolescent happiness. Multicollinearity among independent variables was evaluated using variance inflation factor values, with values exceeding 5 indicating significant multicollinearity. In addition, we aimed to explore the potential mediating roles of anxiety, depression and regular physical exercise in happiness, using the PROCESS procedure for SPSS (version 3.4.1, model 4) developed by Hayes for mediation analysis. Mediating effects were confirmed through bootstrapping with 5000 samples, with a 95% confidence interval that excluded zero indicating significant effects.

### Ethical disclosure

The authors assert that all procedures contributing to this work comply with the ethical standards of the relevant national and institutional committees on human experimentation and with the Helsinki Declaration of 1975, as revised in 2013. All procedures involving human subjects and/or patients were approved by the hospital’s ethics committee, associated with Shandong First Medical University (SWYX: NO2021-312). Before participating in the study, all adolescents and their guardians were informed about the details of the study, and written consent was collected from each participant and his or her guardian.

## Results

### Sociodemographic and clinical characteristics

Table 1 presents the sociodemographic and clinical characteristics of a study sample comprising 392 adolescents. The median age of participants was 15 years, with most aged between 14 and 16 years. The sex distribution showed 37.2% male and 62.8% female participants. Regarding educational attainment, 39.8% attended junior high, and 60.2% were in senior high school. Most adolescents (70.2%) had siblings, whereas 29.8% were only children. Residential data indicated that 72.4% of the sample resided in urban areas, and 27.6% lived in rural areas. Parental marital quality varied, with 45.7% reporting good, 39.5% fair and 14.8% poor relationships. The general health condition of the adolescents was predominantly fair (65.1%), with fewer reporting good (20.7%) or poor (14.3%) health.

**Table 1:** Sociodemographic and clinical characteristics of adolescents

Variables	Overall (*n* = 392)
Age, years (IQR)	15 (14, 16)
Sex, *n* (%)	
Male	146 (37.2)
Female	246 (62.8)
Education level, *n* (%)	
Junior high school	156 (39.8)
Senior high school	236 (60.2)
Siblings, *n* (%)	
Only child	117 (29.8)
At least one sibling	275 (70.2)
Residence, *n* (%)	
Rural	108 (27.6)
Urban	284 (72.4)
Quality of parents’ marriages, *n* (%)	
Good	179 (45.7)
Fair	155 (39.5)
Poor	58 (14.8)
General health condition, *n* (%)	
Good	81 (20.7)
Fair	255 (65.1)
Poor	56 (14.3)
Regular physical exercise, *n* (%)	
Yes	133 (33.9)
No	259 (66.1)
Regular diet, *n* (%)	
Yes	249 (63.5)
No	143 (36.5)
History of mental illness, *n* (%)	
Yes	32 (8.2)
No	360 (91.8)
History of somatic disease, *n* (%)	
Yes	38 (9.7)
No	354 (90.3)
GAD-7 score (IQR)	13 (9, 17)
PHQ-9 score (IQR)	18 (13, 22)
Happiness score (IQR)	3 (1, 5)

IQR, interquartile range; PHQ-9, nine-item Patient Health Questionnaire; GAD-7, seven-item Generalized Anxiety Disorder scale.

Regarding physical exercise and dietary habits, 33.9% of the adolescents engaged in regular physical activity, whereas 66.1% did not; and 63.5% of the participants maintained a regular diet, whereas 36.5% did not. The prevalence of mental illness in the sample was relatively low at 8.2%, with a high percentage (91.8%) reporting no history of mental health problems. A similar pattern was observed for somatic diseases, with 9.7% reporting such conditions and 90.3% reporting none. In the psychological assessments, participants had a median GAD-7 score of 13 (interquartile range (IQR): 9, 17) and PHQ-9 score of 18 (IQR: 13, 22), suggesting moderate levels of anxiety and depression symptoms, respectively. For the happiness index, rated on an 11-point scale, participants had a median score of 3 (IQR: 1, 5), indicating a moderate sense of overall happiness.

### Results of the Spearman correlation analysis

Table 2 presents the Spearman correlations between various sociodemographic and clinical characteristics of adolescents and their reported levels of happiness. All results that are reported as statistically significant have passed Bonferroni correction (*P* < 0.05/13 = 0.004). The age of participants showed a slight negative correlation with happiness (*r* = −0.08, *P* = 0.12), but there was no significant association. Similarly, sex (*r* = 0.09, *P* = 0.07) and education level (*r* = −0.05, *P* = 0.32) also demonstrated negligible correlations with happiness. Notably, the quality of parents’ marriages showed a significant positive correlation with adolescent happiness (*r* = 0.18, *P* = 0.0004). General health condition (*r* = 0.30, *P* < 0.0001), regular physical exercise (*r* = 0.28, *P* < 0.0001) and regular diet (*r* = 0.27, *P* < 0.0001) also showed strong positive correlations with happiness, indicating that better health practices and conditions are associated with higher happiness levels.

**Table 2:** Spearman correlations of sociodemographic and clinical characteristics with happiness in adolescents

Variables	Happiness	
	*r*	*P*
Age	−0.08	0.12
Sex	0.09	0.07
Education level	−0.05	0.32
Siblings	−0.06	0.24
Residence	−0.05	0.33
Quality of parents’ marriages	0.18*	0.0004
General health condition	0.30*	<0.0001
Regular physical exercise	0.28*	<0.0001
Regular diet	0.27*	<0.0001
History of mental illness	−0.03	0.58
History of somatic disease	−0.09	0.10
GAD-7	−0.37*	<0.0001
PHQ-9	−0.47*	<0.0001

PHQ-9, nine-item Patient Health Questionnaire; GAD-7, seven-item Generalized Anxiety Disorder scale. *P-value remained significant after Bonferroni correction (*P* < 0.05/13 = 0.004).

Conversely, the presence of mental illness did not show a significant association with happiness (*r* = −0.03, *P* = 0.58), and nor did the presence of a somatic disease (*r* = −0.09, *P* = 0.10). In addition, anxiety and depression scores (measured by GAD-7 and PHQ-9, respectively), showed strong negative correlations with happiness (GAD-7: *r* = −0.37, *P* < 0.0001; PHQ-9: *r* = −0.47, *P* < 0.0001), suggesting that higher levels of anxiety and depression are associated with lower levels of happiness.

### Results of the stepwise linear regression analysis

Table 3 shows the results of the stepwise linear regression analysis to assess the impact of sociodemographic and clinical characteristics on happiness among adolescents. Depression severity as measured by the PHQ-9 had a significant negative impact on happiness, with an unstandardised coefficient (*B*) of −0.17 and a standardised coefficient (*β*) of −0.40 (*t* = −8.52, *P* < 0.0001), suggesting a strong inverse relationship. Conversely, regular physical exercise appeared to be a significant positive predictor of happiness, with a *B* of 0.73 and a *β* of 0.13 (*t* = 2.75, *P* = 0.006). Quality of parents’ marriage was also a positive predictor, showing a *B* of 0.47 and a *β* of 0.12 (*t* = 2.78, *P* = 0.006). Similarly, maintaining a regular diet had a positive association with happiness, with a *B* of 0.57 and a *β* of 0.10 (*t* = 2.16, *P* = 0.03).

**Table 3:** Stepwise linear regression analysis of sociodemographic characteristics, clinical characteristics and happiness in adolescents

Variables	Unstandardised coefficients		Standardised coefficients	*t*	*P*	VIF	95% CI		*R* ^2^	Adj. *R* ^2^
	*B*	s.e.	*β*				Lower bound	Upper bound		
PHQ-9	−0.17	0.02	−0.40	−8.52	<0.0001	1.21	−0.21	−0.13	0.28	0.28
Regular physical exercise	0.73	0.27	0.13	2.75	0.006	1.12	0.21	1.25		
Quality of parents’ marriages	0.47	0.17	0.12	2.78	0.006	1.02	0.14	0.80		
Regular diet	0.57	0.27	0.10	2.16	0.03	1.16	0.05	1.10		

VIF, variance inflation factor; adj., adjusted; PHQ-9, nine-item Patient Health Questionnaire.

Variance inflation factor values for all variables were well below the commonly used threshold of 5, indicating no significant multicollinearity concerns. The model’s overall fit was robust, explaining 28% of the variance in happiness (*R* ^2^ = 0.28); the adjusted *R* ^2^ was also 0.28, indicating the significant explanatory power of these variables.

### Results of the mediation analysis

To further understand the mediating effects of the variables, we assessed the significance of each indirect effect by introducing bootstrapping. Specifically, 5000 bootstrap samples were taken, and 95% confidence intervals were calculated. The corresponding 95% confidence intervals for each path did not contain zero, indicating that the total, direct and indirect effects were significant. Tables 4–6 present detailed results for the total, direct and indirect effects of regular physical exercise and psychological (depression and anxiety) symptoms on happiness among adolescents, based on a sample size of 392.

**Table 4:** Total, direct and indirect effects of regular physical exercise on happiness through depressive symptoms (*N* = 392)

Effect	Estimate	Boot s.e.	Total effect percentage	Boot 95% CI		*t*	*P*
				Lower	Upper		
Total effect	1.23	0.28	100%	0.68	1.78	4.38	<0.0001
Direct effect	0.73	0.26	59.3%	0.21	1.25	2.75	0.006
Indirect effect	0.50	0.14	40.7%	0.25	0.80	–	–

Based on 5000 bootstrap samples; 95% bias-corrected confidence interval.

**Table 5:** Total, direct and indirect effects of regular physical exercise on happiness through anxiety symptoms (*N* = 392)

Effect	Estimate	Boot s.e.	Total effect percentage	Boot 95% CI		*t*	*P*
				Lower	Upper		
Total effect	1.23	0.28	100%	0.68	1.78	4.38	<0.0001
Direct effect	0.91	0.27	74.0%	0.38	1.44	3.38	0.0008
Indirect effect	0.32	0.11	26.0%	0.12	0.57	–	–

Based on 5000 bootstrap samples; 95% bias-corrected confidence interval.

**Table 6:** Total, direct and indirect effects of regular physical exercise (X) on happiness (Y) through anxiety symptoms (M1) and depression symptoms (M2) (*N* = 392)

Effect	Estimate	s.e.	Percentage of total effects	95% CI		*t*	*P*
				Lower	Upper		
Total effects	1.59	0.28	100%	1.03	2.15	5.61	<0.0001
Direct effect	0.84	0.27	52.83%	0.32	1.37	3.19	0.0016
Total indirect effect	0.75	0.16	47.17%	0.45	1.09	–	–
Indirect effect (X→M1→Y)	0.09	0.10	5.66%	−0.09	0.29	–	–
Indirect effect (X→M1→M2→Y)	0.29	0.10	18.24%	0.12	0.50	–	–
Indirect effect (X→M2→Y)	0.37	0.12	23.27%	0.17	0.64	–	–

Based on 5000 bootstrap samples; 95% bias-corrected confidence interval.

#### Effects of physical exercise on happiness through partial mediation by depressive symptoms

**Fig. 1:**
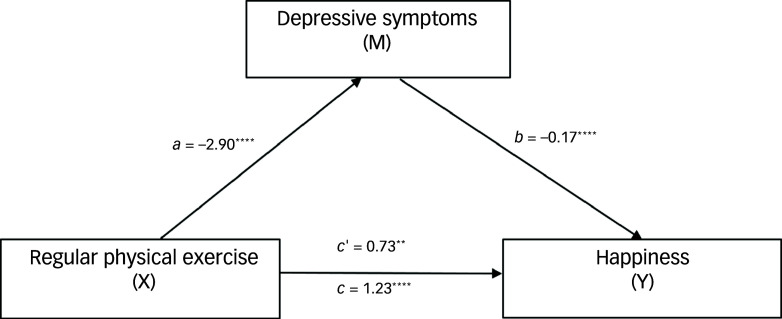
Mediation of effects among regular physical exercise, happiness and depressive symptoms (*N* = 392). The total effect of exercise on happiness was robust (estimate = 1.23, *P* < 0.0001), indicating that physical exercise significantly enhances happiness levels. The direct effect of exercise, without consideration of depressive symptoms, contributed 59.3% to happiness (estimate = 0.73, *P* = 0.006). The indirect effect, operating through the reduction of depressive symptoms, accounted for 40.7% (estimate = 0.50, boot 95% CI: 0.25 to 0.80). *a*, the effect of the independent variable (exercise) on the mediator (depressive symptoms); *b*, the effect of the mediator (depressive symptoms) on the dependent variable (happiness); *c*, the total effect of the independent variable (exercise) on the dependent variable (happiness), including both direct and indirect effects; *c*’, the direct effect of the independent variable (exercise) on the dependent variable (happiness), after accounting for the mediator (depressive symptoms). **P < 0.01; ****P < 0.0001.

Table 4 presents the total, direct and indirect effects of regular physical exercise on happiness, mediated by depressive symptoms (Fig. 1). The total effect of exercise on happiness was robust (estimate = 1.23, boot s.e. = 0.28, boot 95% CI: 0.68 to 1.78, *t* = 4.38, *P* < 0.0001), indicating that physical exercise significantly enhances happiness levels. The direct effect of exercise, without considering depressive symptoms, contributed 59.3% to this happiness (estimate = 0.73, boot s.e. = 0.26, boot 95% CI: 0.21 to 1.25, *t* = 2.75, *P* = 0.006), whereas the indirect effect, operating through a reduction in depressive symptoms, accounted for 40.7% (estimate = 0.50, boot s.e. = 0.14, boot 95% CI: 0.25 to 0.80).

#### Effects of physical exercise on happiness through partial mediation by anxiety symptoms

**Fig. 2:**
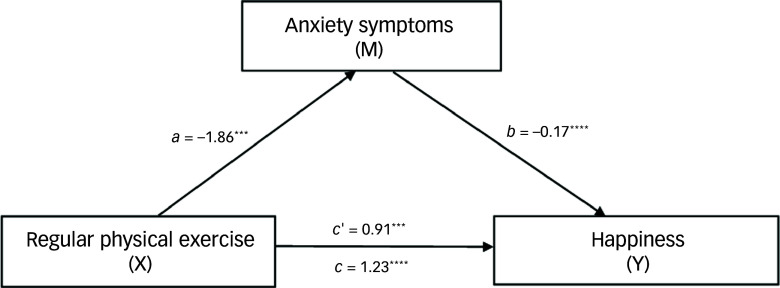
Mediation of effects among regular physical exercise, happiness and anxiety symptoms (*N* = 392). The total effect of exercise on happiness was robust (estimate = 1.23, *P* < 0.0001), indicating that physical exercise significantly enhances happiness levels. The direct effect accounted for a larger portion of happiness (74.0%; estimate = 0.91, *P* = 0.0008). The indirect effect via anxiety symptoms contributed 26.0% to happiness (estimate = 0.32, boot 95% CI: 0.12 to 0.57). *a*, the effect of the independent variable (exercise) on the mediator (anxiety symptoms); *b*, the effect of the mediator (anxiety symptoms) on the dependent variable (happiness); *c*, the total effect of the independent variable (exercise) on the dependent variable (happiness), encompassing both direct and indirect effects; *c’*, the direct effect of the independent variable (exercise) on the dependent variable (happiness), controlling for the mediator (anxiety symptoms). ***P < 0.001; ****P < 0.0001.

Table 5 shows results for mediation by anxiety symptoms. The total effect was consistent with the findings for mediation by depression shown in Table 4 (Fig. 2). However, the direct effect accounted for a greater proportion of happiness (74.0%; estimate = 0.91, boot s.e. = 0.27, boot 95% CI: 0.38 to 1.44, *t* = 3.38, *P* = 0.0008), whereas the indirect effect via anxiety symptoms contributed 26.0% to happiness (estimate = 0.32, boot s.e. = 0.11, boot 95% CI: 0.12 to 0.57).

#### Multiple chain-mediated effects of regular physical activity on happiness through anxiety and depressive symptoms

**Fig. 3:**
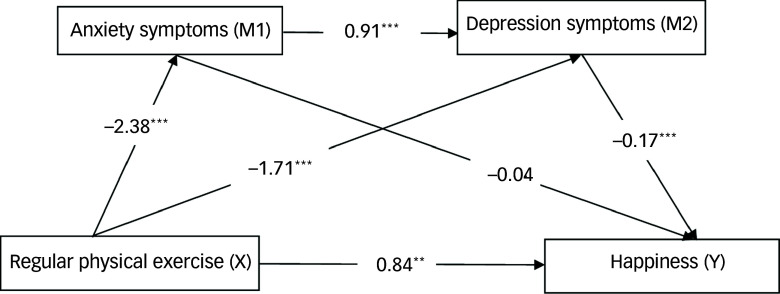
Multiple chain-mediated effects of regular physical activity on well-being through anxiety and depressive symptoms (*N* = 392). The total effect of exercise on happiness was estimated to be 1.59, with a standard error of 0.28, indicating a significant positive relationship (boot 95% CI: 1.03 to 2.15; *t* = 5.61, *P* < 0.0001). The direct effect of exercise on happiness was 0.84, representing 52.83% of the total effect; this effect was also statistically significant (boot 95% CI: 0.32 to 1.37; *t* = 3.19, *P* = 0.0016). The total indirect effect of exercise on happiness was estimated at 0.75, accounting for 47.17% of the total effect (boot 95% CI: 0.45 to 1.09). Among the specific indirect effects, the pathway from exercise to happiness through anxiety symptoms (X→M1→Y) was minimal, with an estimate of 0.09 (5.66% of total effects); this effect was not statistically significant (boot 95% CI: −0.09 to 0.29). By contrast, the indirect effect through both anxiety and depression (X→M1→M2→Y) was estimated to be 0.29, representing 18.24% of the total effects; this effect was statistically significant (boot 95% CI: 0.12 to 0.50). In addition, the effect of exercise on happiness through depression alone (X→M2→Y) was estimated at 0.37, accounting for 23.27% of the total effects; this was also statistically significant (boot 95% CI: 0.17 to 0.64). These findings indicate that although physical exercise directly enhances happiness, it also significantly influences happiness indirectly, primarily through depression symptoms. **P< 0.01; ***P< 0.001.

Table 6 presents the total, direct and indirect effects of regular physical exercise on happiness (Fig. 3). The total effect of exercise on happiness was estimated to be 1.59, indicating a strong positive influence, with an s.e. of 0.28 and a 95% CI ranging from 1.03 to 2.15 (*t* = 5.61, *P* < 0.0001). The direct effect of exercise on happiness was 0.84, which constituted 52.83% of the total effect, and this effect was statistically significant (95% CI: 0.32 to 1.37, *t* = 3.19, *P* = 0.0016).

The total indirect effect of exercise on happiness, mediated by anxiety symptoms and depression symptoms, was estimated to be 0.75, accounting for 47.17% of the total effect (95% CI: 0.45 to 1.09). Among the specific indirect effects, the pathway from exercise to happiness through anxiety (X→M1→Y) had a small effect size of 0.09 (5.66% of total effects), which was not statistically significant (95% CI: −0.09 to 0.29). By contrast, the indirect effect through both anxiety and depression (X→M1→M2→Y) was estimated to be 0.29 (18.24% of total effects) and was statistically significant (95% CI: 0.12 to 0.50). Last, the effect of exercise on happiness through depression alone (X→M2→Y) was estimated to be 0.37 (23.27% of total effects), which was also statistically significant (95% CI: 0.17 to 0.64). Overall, these findings suggest that although physical exercise has a significant direct impact on happiness, it also exerts substantial indirect effects through depression symptoms. This finding highlights the complex interplay among these variables.

#### Effects of anxiety symptoms on happiness through complete mediation by depressive symptoms

**Fig. 4:**
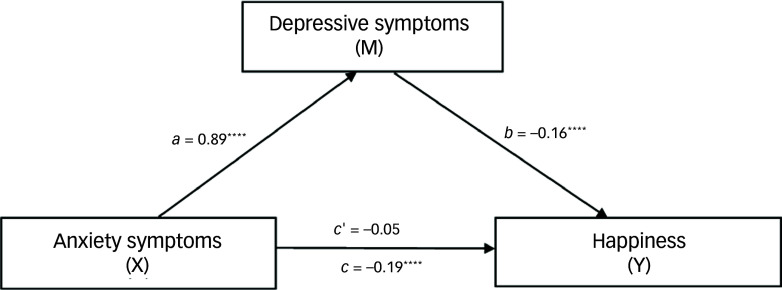
Mediation of effects among anxiety symptoms, happiness and depression symptoms (*N* = 392). The total effect was negative (estimate = −0.19, *P* < 0.0001), indicating that anxiety symptoms significantly detract from happiness. The direct effect was less influential and not statistically significant (estimate = −0.05, *P* = 0.18). The substantial indirect effect through depressive symptoms significantly exacerbated the negative impact on happiness, accounting for 73.7% of the total effect (estimate = −0.14, boot 95% CI: −0.20 to −0.08). *a*, the effect of the independent variable (anxiety symptoms) on the mediator (depressive symptoms); *b*, the effect of the mediator (depressive symptoms) on the dependent variable (happiness); *c*, the total effect of the independent variable (anxiety symptoms) on the dependent variable (happiness), which includes both direct and indirect effects; *c’*, the direct effect of the independent variable (anxiety symptoms) on the dependent variable (happiness), while controlling for the mediator (depressive symptoms). ****P < 0.0001.

Table 7 shows the results of the analysis of the impact of anxiety symptoms on happiness, mediated by depressive symptoms (Fig. 4). The total effect was negative (estimate = −0.19, boot s.e. = 0.02, boot 95% CI: −0.24 to −0.14, *t* = −7.63, *P* < 0.0001), indicating that anxiety symptoms significantly detract from happiness. The direct effect was less influential and not significant (estimate = −0.05, boot s.e. = 0.04, boot 95% CI: −0.12 to 0.02, *t* = −1.34, *P* = 0.18). The substantial indirect effect through depressive symptoms significantly exacerbated the negative impact on happiness, accounting for 73.7% of the total effect (estimate = −0.14, boot s.e. = 0.03, boot 95% CI: −0.20 to −0.08).

**Table 7:** Total, direct and indirect effects of anxiety symptoms on happiness through depression symptoms (*N* = 392)

Effect	Estimate	Boot s.e.	Total effect percentage	Boot 95% CI		*t*	*P*
				Lower	Upper		
Total effect	−0.19	0.02	100%	−0.24	−0.14	−7.63	<0.0001
Direct effect	−0.05	0.04	26.3%	−0.12	0.02	−1.34	0.18
Indirect effect	−0.14	0.03	73.7%	−0.20	−0.08	–	–

Based on 5000 bootstrap samples; 95% bias-corrected confidence interval.

## Discussion

To our knowledge, this is the first study conducted among adolescents seeking psychological services that explores risk factors related to happiness, with a particular focus on the mediating effects of anxiety and depression on adolescent happiness. This comprehensive assessment of 392 adolescents showed that parental marital quality, emotional conditions, physical exercise and dietary habits have significant correlations with and predictive value for happiness. The mediation analysis showed that regular physical exercise not only directly enhances adolescents’ happiness but also indirectly improves it by alleviating the negative effects of depressive and anxiety symptoms. Conversely, higher levels of anxiety and depression correspond to lower levels of happiness, with depressive symptoms acting as a complete mediator between anxiety symptoms and reduced happiness. These findings suggest that it is crucial for interventions aimed at improving adolescent happiness to address both physical and psychological health issues.

Our comprehensive assessment of 392 adolescents highlighted significant relationships of parental marital quality and dietary habits with adolescent happiness. The previous literature supports these findings, with studies suggesting that stable family environments and healthy lifestyle choices are important for adolescent happiness. For instance, compared with their counterparts from homes with high marital quality, children of parents with lower marital quality are more prone to internalising and externalising problems and more likely to have poorer health, experience inferior home environments, and achieve lower scores in mathematics and vocabulary.^29^ These results indicate the significance of a nurturing home environment for mental health during adolescence. Moreover, research indicates a strong link between dietary quality and increased subjective happiness in children and adolescents, primarily owing to enhanced emotional happiness and social acceptance, suggesting that optimal nutrition is key to fostering overall mental health from an early age.^30^ This underscores the importance of promoting healthy eating habits to support both mental and physical happiness in the younger population.

The present study demonstrated that the consequences of anxiety and depression for adolescent happiness are profound. These findings resonate with those of previous research indicating strong inverse relationships of mental health disorders such as anxiety and depression with happiness,^31^ i.e. the higher the levels of these mental health problems, the lower the happiness levels. Research has also found that anxiety and depression adversely affect the happiness of adolescents by lowering their self-esteem and overall life satisfaction. Such conditions tend to create a generalised sense of hopelessness that severely affects adolescents’ quality of life.^32^ These findings suggests that effective therapeutic interventions should target these symptoms to improve happiness.

The present study found serial multiple mediating roles for anxiety symptoms and depressive symptoms in the relationship between regular physical exercise and happiness, consistent with reports of previous studies.^15^ The results of the current study also indicate a dual role of physical exercise in improving adolescents’ happiness directly and indirectly by reducing anxiety and depressive symptoms. This also aligns with prior research showing significant correlations of physical activity with both reduced psychological distress (e.g. depression, stress, negative affect and overall psychological distress) and enhanced psychological happiness (e.g. self-image, life satisfaction, happiness and general psychological happiness). Moreover, higher levels of sedentary behaviour are associated with increased psychological distress (e.g. depression) and diminished psychological happiness (e.g. life satisfaction and happiness) in children and adolescents.^33^ The consequent dual benefit of exercise underscores the importance of incorporating physical exercise into interventions aimed at improving mental health and overall happiness. However, although previous research suggests that physical activity can positively affect the mental health of children and adolescents, particularly through reducing depression and anxiety, the effects reported have been generally small and inconsistent, with some studies showing minimal or negligible benefits owing to weak intervention designs. There is thus a clear need for more rigorous research in this area.^34^ Physical activity is not a straightforward behaviour but the outcome of a complex interplay of physiological, psychological and social factors.^35^ It is a multifaceted behaviour that varies across dimensions including intensity, duration, frequency and type, all of which can be assessed through either self-reports or objective methods.^36^ The diversity of measurement techniques introduces potential biases and inconsistencies in findings; therefore, these factors should be considered carefully in research. Furthermore, socioeconomic and demographic variables significantly influence physical activity behaviours and must be meticulously controlled to ensure the validity and reliability of research outcomes.^37^

The results of this study resonate with the theoretical framework proposed by McGreal and Joseph,^38^ which conceptualised depression and happiness as points on a continuum rather than separate, independent states. Their ‘Depression-Happiness Scale’ emphasised the interrelatedness of these emotional states – a concept that the current study’s findings confirm. We found that regular physical exercise could alleviate symptoms of anxiety and depression, promoting an upward movement along the depression–happiness continuum and thereby enhancing overall happiness. This finding also aligns with the perspective of McGreal and Joseph that emotional happiness is a dynamic, continuous construct. The observed increase in happiness scores in the present study provides empirical support for the notion that interventions such as regular exercise that targeting reduction of negative emotions can simultaneously promote positive emotional states. In addition, our results contribute to a deeper understanding of the complex relationships among physical exercise, anxiety and depression, and enhanced happiness. By identifying anxiety and depression as sequential mediators, we demonstrate the need for integrated intervention strategies that address these interrelated emotional states simultaneously. The emotional happiness continuum model proposed by McGreal and Joseph, combined with the current empirical findings, provides a strong framework for designing targeted mental health interventions that fully leverage the psychological benefits of regular physical exercise.

Research examining the indirect effects of anxiety and depression on happiness, particularly in relation to regular exercise, has revealed an interesting pattern. Although both anxiety and depression symptoms demonstrate significant indirect effects when analysed separately, only the indirect effect of depression remains significant when they are analysed as serial mediators. This finding suggests that in cases of comorbidity, the impact of depressive symptoms may overshadow that of anxiety symptoms. This could be because during prolonged stressful situations, such as the COVID-19 pandemic, depressive symptoms often become more dominant and severe.^39^ This ‘masking effect’ indicates a need for careful consideration in psychological interventions, ensuring that anxiety symptoms are not overlooked when treating individuals with comorbid depression. The implications for clinical practice are significant. Interventions should be designed to recognise and address the dominant depressive symptoms while not neglecting the potential underlying anxiety. Comprehensive assessments that evaluate both anxiety and depressive symptoms are crucial for accurate diagnosis and effective treatment-planning.

The key finding of the present study was that depressive symptoms serve as a complete mediator between anxiety symptoms and sense of happiness, indicating that the primary effect of anxiety symptoms on happiness is exerted through depressive symptoms. Specifically, anxiety symptoms in adolescents tend to lead to depressive symptoms, which then fully mediate the effect of anxiety on the reduction in happiness. This observation is consistent with previous literature confirming the intricate relationship between anxiety and depression in children and adolescents and indicating that anxiety often precedes depression.^40^ Importantly, these findings highlights the preventive potential of timely interventions when anxiety symptoms appear, to prevent the development of depressive symptoms and maintain a greater sense of happiness. Conversely, our results also emphasise the importance of intervening in cases of depressive symptoms, as this can effectively mitigate the broad psychological impact of anxiety, thereby improving the happiness of adolescents. Unfortunately, there have not been any previous studies on the mediating role of depressive symptoms in the relationship between adolescent anxiety symptoms and happiness. Thus, we cannot directly compare our results with those of previous research; however, we may conclude that the complete mediating role of depressive symptoms is crucial for adolescent happiness.

This study had several limitations. First, the reliance on self-reported data to assess factors including happiness, physical exercise, dietary habits, and symptoms of anxiety and depression may have introduced bias. Self-report measures can be subject to inaccuracies owing to respondents’ desires to present themselves favourably or to misunderstandings of the questions. Second, the cross-sectional design of the study limited its ability to establish causality between the examined factors (e.g. parental marital quality, physical exercise, dietary habits) and adolescent happiness. Longitudinal designs are preferable for understanding the dynamics of such relationships over time. Third, the findings may not be generalisable to all adolescent populations, especially if the sample lacked diversity in terms of socioeconomic status, ethnicity or geographical location. Different cultural backgrounds and environmental contexts could influence the impact of marital quality, lifestyle choices and mental health on adolescent happiness. Fourth, the strict exclusion criteria applied in this study, although essential for controlling confounding variables and ensuring internal validity, may limit the generalisability of the findings to broader populations. Finally, this study highlights the limitation of using binary measures for physical activity and indicates a need for more nuanced methodologies to be used in future research to encompass the complete spectrum of activity behaviours.

Regarding future clinical and research implications, the present results call for targeted interventions that address both the physical and psychological dimensions of health to foster resilience against mental health challenges and enhance happiness among adolescents. Given the limitations of self-report bias, cross-sectional design and potential issues with generalisability, future research should incorporate longitudinal studies and a more diverse population to deepen our understanding of these relationships and verify our findings across different contexts.

## Data Availability

The data supporting this study are available from the corresponding author, Y.D., upon reasonable request.
